# Loss of *Parp-1 *affects gene expression profile in a genome-wide manner in ES cells and liver cells

**DOI:** 10.1186/1471-2164-8-41

**Published:** 2007-02-07

**Authors:** Hideki Ogino, Tadashige Nozaki, Akemi Gunji, Miho Maeda, Hiroshi Suzuki, Tsutomu Ohta, Yasufumi Murakami, Hitoshi Nakagama, Takashi Sugimura, Mitsuko Masutani

**Affiliations:** 1 ADP-ribosylation in Oncology Project, National Cancer Center Research Institute, 1-1, Tsukiji 5-chome, Chuo-ku, Tokyo 104-0045, Japan; 2 Biochemistry Division, National Cancer Center Research Institute, 1-1, Tsukiji 5-chome, Chuo-ku, Tokyo 104-0045, Japan; 3 Department of Biological Science & Technology, Faculty of Industrial Science & Technology, Tokyo University of Science, 2641, Yamazaki, Noda, Chiba 278-8510, Japan; 4 Chugai Pharmaceutical Co Ltd., 1-135, Komakado, Gotemba, Shizuoka, 412-0038, Japan; 5 Center for Medical Genomics, National Cancer Center Research Institute, 1-1, Tsukiji 5-chome, Chuo-ku, Tokyo 104-0045, Japan

## Abstract

**Background:**

Many lines of evidence suggest that poly(ADP-ribose) polymerase-1 (Parp-1) is involved in transcriptional regulation of various genes as a coactivator or a corepressor by modulating chromatin structure. However, the impact of *Parp-1*-deficiency on the regulation of genome-wide gene expression has not been fully studied yet.

**Results:**

We employed a microarray analysis covering 12,488 genes and ESTs using mouse *Parp-1*-deficient (*Parp-1*^-/-^) embryonic stem (ES) cell lines and the livers of *Parp-1*^-/- ^mice and their wild-type *(Parp-1*^+/+^) counterparts. Here, we demonstrate that of the 9,907 genes analyzed, in *Parp-1*^-/- ^ES cells, 9.6% showed altered gene expression. Of these, 6.3% and 3.3% of the genes were down- or up-regulated by 2-fold or greater, respectively, compared with *Parp-1*^+/+ ^ES cells (*p *< 0.05). In the livers of *Parp-1*^-/- ^mice, of the 12,353 genes that were analyzed, 2.0% or 1.3% were down- and up-regulated, respectively (*p *< 0.05). Notably, the number of down-regulated genes was higher in both ES cells and livers, than that of the up-regulated genes. The genes that showed altered expression in ES cells or in the livers are ascribed to various cellular processes, including metabolism, signal transduction, cell cycle control and transcription. We also observed expression of the genes involved in the pathway of extraembryonic tissue development is augmented in *Parp-1*^-/- ^ES cells, including *H19*. After withdrawal of leukemia inhibitory factor, expression of *H19 *as well as other trophoblast marker genes were further up-regulated in *Parp-1*^-/- ^ES cells compared to *Parp-1*^+/+ ^ES cells.

**Conclusion:**

These results suggest that *Parp-1 *is required to maintain transcriptional regulation of a wide variety of genes on a genome-wide scale. The gene expression profiles in *Parp-1*-deficient cells may be useful to delineate the functional role of Parp-1 in epigenetic regulation of the genomes involved in various biological phenomena.

## Background

Poly(ADP-ribose) polymerase-1 (Parp-1) is a nuclear protein that catalyzes the transfer of ADP-ribose units to various nuclear proteins as a post-translational modification [1]. Poly (ADP-ribose) is a highly negatively charged molecule and poly (ADP-ribosylation) of chromatin-bound proteins including histone may change the interaction of the modified proteins with DNA or other proteins. A 'histone shuttle model' proposed by Althaus et al. can explain the dynamic changes of chromatin structure through histone replacement induced by Parp-1 activation [2]. Accumulating evidence suggests that under *Parp-1 *deficiency, transcriptional regulation, cell differentiation, and tumorigenesis are substantially affected. For example, Parp-1 is involved in the regulation of *Reg3 *gene [3] as a transcription factor. As a co-activator, Parp-1 plays a role in the regulation of ligand-induced transactivation of ecdysone receptor [4], and in the transcriptional control of the target genes by AP-2 [5], and by MYB [6]. As a co-repressor, Parp-1 regulates the expression of RXR-regulated genes [7] and also plays an auto-regulatory role in the transcription of the *Parp-1 *gene itself [8]. Parp-1 also modulates the activity of the transcription factor NF-κB and consequently, the expression of NF-κB-dependent genes, including *inducible nitric oxide synthetase *(*iNOS*) [9]. The expression of nearly 1% of the genes, including those involved in cell cycle control and DNA replication was affected in exon 2 disrupted *Parp-1*^-/- ^mouse embryonic fibroblasts (EF cells) [10]. *Parp*-deficient *Drosophila *showed attenuation of gene expression located in puff loci and also lost puff formation, suggesting a role for Parp in the induction of genes located at specific chromosomal loci [11].

Recent studies further suggest that Parp-1 is involved in the regulation of dynamic changes of gene expression induced by specific stimuli. Parp-1 is associated with transcriptionally repressed chromatin domains, which do not overlap with the regions where histone H1 is located [12]. NAD-dependent alteration of chromatin structure through Parp-1 auto-modification was demonstrated to lead to activation of estrogen induced estrogen receptor dependent transcription [12]. In addition, the PARP inhibitor, 3-aminobenzamide induced hypermethylation of the *Htf9 *gene, suggesting the presence of a negative correlation between poly(ADP-ribosylation) and DNA methylation [13]. In spite of the above evidence, how Parp-1 is involved in the epigenetic regulation and functions in the maintenance of basal gene expression profiles of cells are not well understood.

We previously reported induction of the trophoblast lineage in exon 1 disrupted *Parp-1*^-/- ^ES cells during teratocarcinoma-like tumor formation [14], as well as *in vitro *culture [15]. Simultaneous induction of several trophoblast marker genes, including *placental lactogen I *and *II, proliferin *and *Tpbp *(*4311*) in *Parp-1*^-/- ^ES cells took place without any stimulus during trophoblast induction [15]. We therefore considered that ES cells as well as tissues in live mice might be good material in which to study the effects of *Parp-1 *deficiency on a basal level of gene expression, namely epigenetic regulation, at the genome-wide level. In this study, global gene expression profiles were studied in exon 1 disrupted *Parp-1*^-/- ^ES cells as well as in the livers of mice.

## Results and discussion

### Gene expression profile in *Parp-1*^-/- ^ES cells

**Figure 1:**
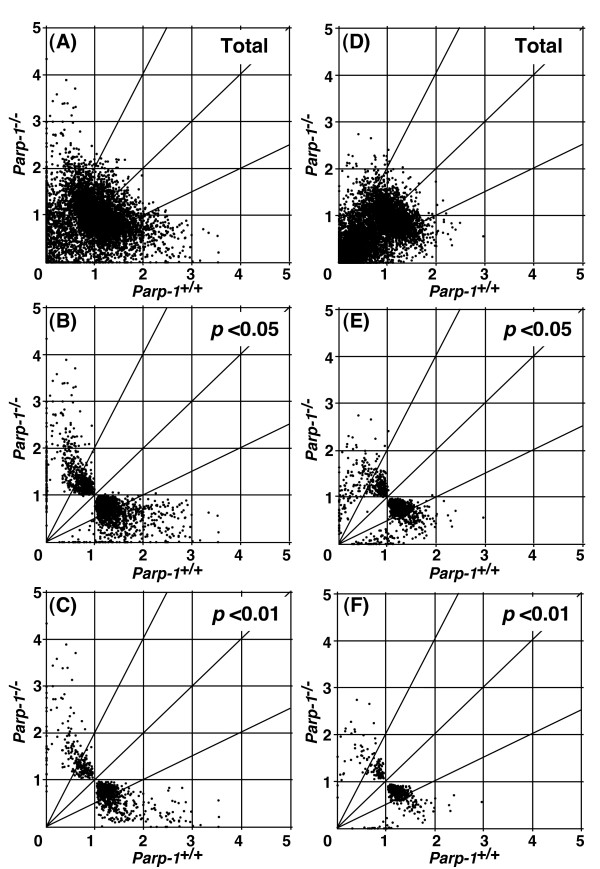
**Effect of *Parp-1 *deficiency on gene expression**. Gene expression data from microarray analyses are plotted for *Parp-1*^-/- ^versus wild-type (*Parp-1*^+/+^) ES cell lines (A-C) or the livers (D-F). Horizontal and vertical axes represent expression levels normalized for an individual gene. Each point represents normalized expression data for an individual gene. The genes that showed standard deviations greater than 2.0 in the normalized data of both genotypes (A and D) were excluded and gene lists were constructed with *p *< 0.05 (B and E), or *p *< 0.01 (C and F).

A comparison of the basal gene expression profiles in *Parp-1*^-/- ^*ES cells *to their wild-type (*Parp-1*^+/+^) counterparts, is presented in Fig. 1A–C and Table 1. We found the expression of (950/9,907) genes, namely 9.6%, was different by at least 2-fold between *Parp-1*^-/- ^and *Parp-1*^+/+ ^ES cells (*p *< 0.05) (Fig. 1B and Table 1). Notably, a larger fraction of the genes, 6.3% (626/9,907) was down-regulated, whereas only 3.3% (324/9,907) of the genes were up-regulated (see Table 1).

**Figure 2:**
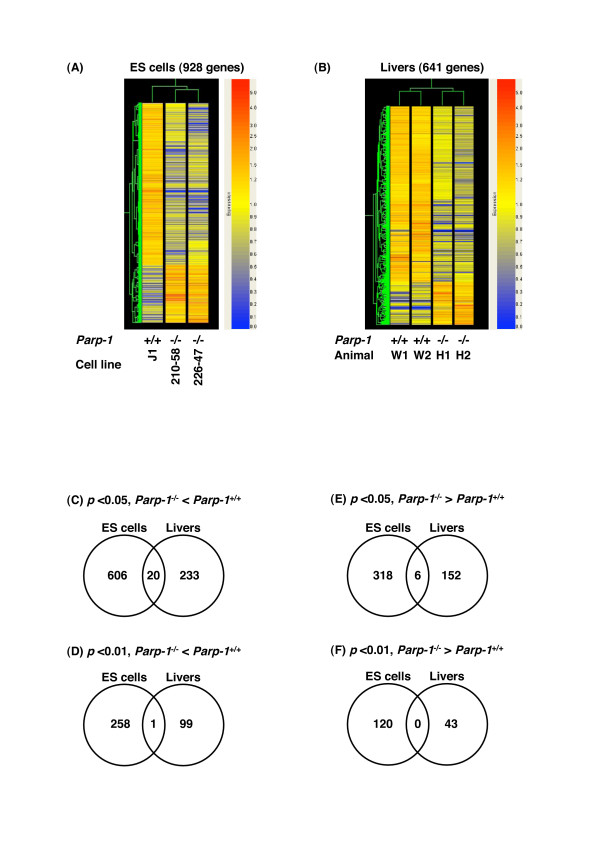
**Comparison of gene expression profiles among cell lines, animals, or cell types**. Heatmaps of gene expression profiles in ES cells (A) and Livers (B). We constructed the heatmaps using the gene lists containing the genes that showed a difference at *p *< 0.01 in ES cells and livers, respectively. Each heatmap is constructed using GeneSpring GX ver. 7.3.1. Numbers of commonly down- (C & D) or up- (E & F) regulated genes between *Parp-1*^-/- ^ES cells and livers. The numbers of the genes were indicated in Venn diagrams. These genes showed the difference with at least 2-fold between *Parp-1*^+/+ ^and *Parp-1*^-/- ^(*p *< 0.05, C & E, or *p *< 0.01, D & F).

We also made the heatmaps using the gene lists containing the 928 genes that showed a difference at *p *< 0.01 in ES cells (Fig. 2A). Although we used independently isolated *Parp-1*^-/- ^ES cell clones, a clear common alteration in the gene expression profile was observed (see Fig. 2A, and Tables 2 and 3).

**Figure 3:**
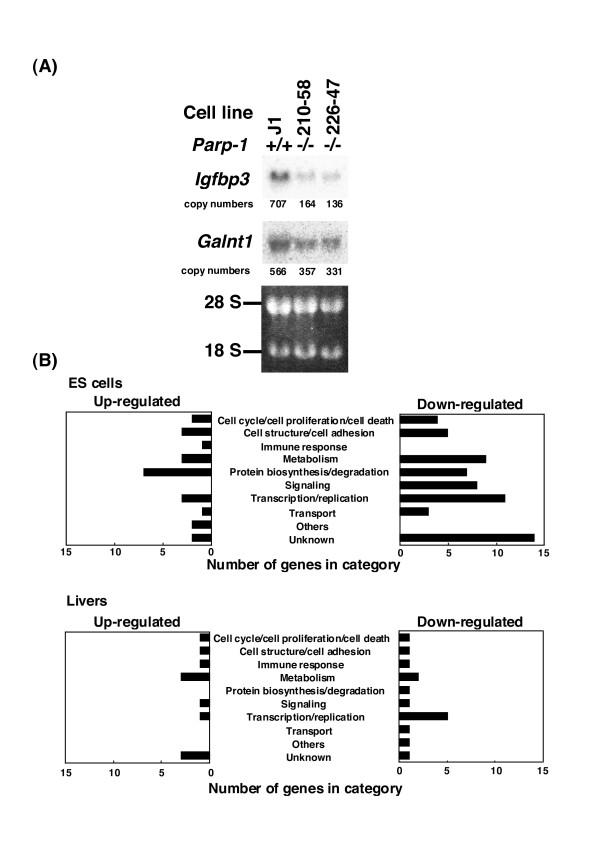
**Confirmation of differentially expressed genes in microarray analysis by northern blot analysis (A), and functional categorization of up- and down-regulated genes (B)**. Ten micrograms of total RNA were used for northern blot analysis in (A). Copy numbers were calculated from the radioactivities of the probe control.

We further selected the genes that showed relatively high expression levels (the "Flag value" in GeneSpring ver. 6.1 of the genes should be either "Present" (high level of expression) or "Marginal" (moderate level of expression) in all six replicates of the genotype within the 928 genes that showed a difference at *p *< 0.01, see Table 1). Among the 86 genes that this analysis identified, there were 62 genes, obviously including the *Parp-1 *(*Adprt1*) gene itself, that were down-regulated and 24 genes up-regulated, as listed in Tables 2 and 3. Reduced expression of *Igfbp3 *(*insulin-like growth factor binding protein 3*) and *Galnt1 *(*polypeptide GalNAc transferase-T1*) in *Parp-1*^-/- ^ES cells was further confirmed by Northern blot analysis (Fig. 3A). These down- and up-regulated genes in *Parp-1*^-/- ^ES cells are involved in a variety of cellular processes, including transcription, metabolism, signaling, immune response, cell structure, and other cellular processes (Fig. 3B, and Tables 2 and 3).

### Gene expression profile of the livers and EF cells

In the livers, 3.3% (411/12,353) of genes showed a significant difference in expression level (*p *< 0.05) between the *Parp-1 *genotypes. In the livers of *Parp-1*^-/- ^mice, 2.0% (253/12,353) of the genes were down-regulated and 1.3% (158/12,353) of the genes were up-regulated (*p *< 0.05). Similar to *Parp-1*^-/- ^ES cells, a higher percentage of the genes, 62% (253/411), were down-regulated and the remaining 38% were up-regulated (Fig. 1D–F, and Table 1). The expression of representative marker genes of the liver, including *albumin *(*Alb1*) and *phosphoenolpyruvate carboxykinase *(*Pepck*) was similarly high in both *Parp-1 *genotypes.

The heatmaps were constructed using the gene lists containing the 641 genes that showed a difference at *p *< 0.01 in livers (Fig. 2B). *Parp-1 *deficiency commonly altered gene expression profiles in the livers of two mice analyzed (Fig. 2B, Table 4). Among 641 genes, we identified 26 genes that showed a relatively high level of expression (genes with "Flag values" of either "Marginal" or "Present" in each genotype) and were altered 2-fold or greater between the *Parp-1*^-/- ^and *Parp-1*^+/+ ^livers (*p *< 0.01) (Table 4). Among them, 15 genes were down-regulated and 11 genes were up-regulated.

In the case of the EF cells, the results obtained from these 3 replicates are shown in Table 1. In *Parp-1*^-/- ^EF cells, 1.7% (216/12,359) and 1.7% (205/12,359) genes were down- and up-regulated, respectively (*p *< 0.05). We were not able to construct gene lists with a *p *value less than *p *< 0.02.

### Comparison of the profiles among different cell types

We compared gene expression profiles between *Parp-1*^-/- ^ES cells and the livers. There were no commonly up- or down-regulated genes in Tables 2, 3, 4, namely in the genes showing relatively high expression levels selected by Flag values, although we observed that 20 genes including *Eif2s2 *(*eukaryotic translation initiation factor 2 subunit 2 beta*), *Parp-1*, and 6 genes were commonly down- and up-regulated in the ES cells and livers (*p *< 0.05), respectively (Fig. 2C–F). There was no gene commonly altered in ES cells, livers, and EFs. Comparison of the affected genes in the ES cells, livers, and EF cells thus revealed that *Parp-1*-deficiency mostly altered the expression level of different sets of genes depending on the cell types.

### Up-regulation of the differentiation pathway to extraembryonic tissues in *Parp-1*^-/- ^ES cells

Among the genes, we found up-regulation of *H19, Sparc, Sox17, and Gata6 *in *Parp-1*^-/- ^ES cells (Table 3). The *H19 *gene has been suggested to regulate differentiation into extraembryonic tissues including trophoblast lineage and extraembryonic endoderms [16-18]. *Sparc, Sox17*, and *Gata6 *are known as marker genes of extraembryonic endoderms [19-21]. Because we previously reported induction of trophoblast lineage in untreated *Parp-1*^-/- ^ES cells during *in vitro *culture, we speculated that a higher level of *H19 *expression in *Parp-1*^-/- ^ES cells may be involved in induction of extraembryonic tissues including trophoblast lineage. The mouse *H19 *gene is located on the distal region of chromosome 7 and encodes the 2.3 kb untranslated transcript, which is maternally expressed, and the *H19 *gene and the *insulin-like growth factor 2 *(*Igf2*) gene are reciprocally imprinted [22].

**Figure 4:**
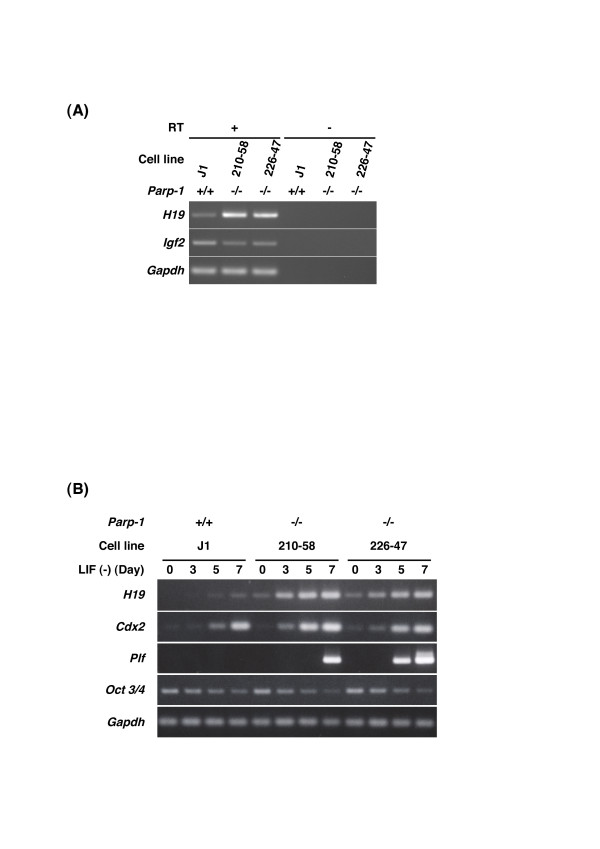
**Semi-quantitative RT-PCR analysis of *H19 *and other extraembryonic marker gene expression in undifferentiatiated ES cells (A) or during differentiation of ES cells after LIF withdrawal (B)**. (A) PCR was carried out using cDNA prepared with (+) or without (-) reverse transcriptase (RT) [see Additional file 1 for primers]. (B) Total RNA was prepared using harvested ES cells 3, 5, and 7 days after removal of LIF. RNA samples prepared from untreated ES cells correspond to Day 0. *Gapdh *(*glyceraldehyde-3-phosphate dehydrogenase*) gene was used as an internal control.

We analyzed expression of *H19 *and *Igf2 *genes in untreated *Parp-1*^-/- ^and *Parp-1*^+/+ ^ES cell lines by semi-quantitative RT-PCR (Fig. 4A). We confirmed that the *H19 *gene is up-regulated, whereas the *Igf2 *gene, which is reciprocally imprinted was slightly down-regulated in both the two *Parp-1*^-/-^ES cell lines.

*H19 *is highly expressed in extraembryonic tissues, including placenta and cells quite similar to the parietal endoderm of extraembryonic lineages, during ES cell differentiation [16]. Because withdrawal of LIF during ES cell culture causes differentiation of ES cells [23,24], we further analyzed expression of the *H19 *gene and other trophoblast marker genes for 7 days after withdrawal of LIF by semi-quantitative RT-PCR. We observed earlier and greater up-regulation of the *H19 *gene in two *Parp-1*^-/- ^ES cells compared to wild-type cells (Fig. 4B). We also observed a higher level of induction of trophoblast stem cell marker gene *caudal-related homeobox 2 *(*Cdx2*) [25]. The induction of trophoblast giant cell marker gene, *proliferin *(*Plf*) [26] was only observed in *Parp-1*^-/- ^ES cell lines (Fig. 4B). In contrast, *POU domain, class 5, transcription factor 1 *(*Oct3/4*) gene, which is a marker gene of undifferentiated ES cells [27], was gradually down-regulated in both genotypes during differentiation, although the expression level of *Oct 3/4 *gene became slightly lower in *Parp-1*^-/- ^than in *Parp-1*^+/+ ^ES cell lines at day 7 after withdrawal of LIF (Fig. 4B).

These results suggest that the potential for differentiation into trophoblasts is increased in ES cells under *Parp-1 *deficiency.

### Possible roles of Parp-1 in global gene expression profiles

Using genome-wide analysis of gene expression in different cell types, we showed that the expression of a number of genes is affected by the loss of *Parp-1 *in both ES cells as well as in the liver. The results suggest that Parp-1 may be involved directly or indirectly in maintenance of their regulation of expression. The genes that showed altered expressions in *Parp-1*^-/- ^ES cells, livers and EF cells are mostly different depending on the cell type, and are not apparently clustered at particular loci on specific chromosomes, and both house-keeping and inducible genes were present in the affected gene lists. Functional categorization of the altered genes in *Parp-1*^-/- ^ES cells and livers showed that these genes are involved in various cellular processes (Fig. 3B). The *Parp-1*^-/- ^and *Parp-1*^+/+ ^ES cells, which we used showed no difference in growth rate [28] and cell-cycle distribution [29], and the karyotype is the same (2*n *= 40) [28]. In mice, we did not observe any differences in body weight nor in the histology of the livers between *Parp-1 *genotypes. Therefore, the differences in gene expression should not be caused indirectly by differences in growth and cell proliferation but might be intrinsic to the absence of Parp-1 molecules. In the case of the EF cells, about 1% of the analyzed genes showed altered levels of expression. We did not observe any genes overlapping between the report on *Parp-1*^-/- ^EF cells disrupted at exon 2 [10], and our present results with the exon 1 disrupted EFs. This may be possibly due to differences in targeting construct, genetic backgrounds or the heterogeneity of EFs.

Accumulating evidence suggests that Parp-1 regulates gene expression by modulating transcriptional factors, including YY1 [30], Oct-1 [31], NF-κB [32], E47 [33], and TEF-1 [34]. In these cases, Parp-1 stimulates loading of these transcriptional factors to cognate target sequences through protein-protein interaction. However, it is noteworthy that the target genes of these transcription factors did not show altered expression in this study. Parp-1 is also able to act as co-activator for retinoic acid receptor (RAR)-mediated transcription of *Rarβ2 *gene [35] and β-catenin/TCF4 complex-dependent transcription [36]. In the case of *RXRα *[7], Parp-1 may act as a co-repressor for ligand-induced gene activation. Again, in this study, the target genes for *Rarβ2 *or *RXRα *genes were not deregulated in *Parp-1*^-/- ^ES cells and in the livers. It is thus suggested that loss of Parp-1 may affect the maintenance of basal expression level of a wide variety of the genes in ES cells and the livers through different mechanisms from the regulation involving these transcription factors.

In addition, PARP-1 binds to the scaffold/matrix attachment region (S/MARs) containing partially unwound AT-rich sequences that form local non-B structures [37]. PARP-1 binds to other non-B DNA structures including hairpin, cruciform, and loop, and is catalytically activated [38]. The variations of gene promoter/enhancer structure and Parp-1 binding and recruitment in different cell types may be possibly related to the observed differences in the effect of *Parp-1 *deficiency on expression profiles.

Since PARP inhibitors are shown to cause hypermethylation of particular genes [13], loss of Parp-1 may possibly cause local changes in DNA methylation pattern during DNA replication and may further affect histone acetylation or methylation, thereby causing genome wide alteration of gene expression after rounds of cell division. In this context, it is notable that similar to the case of *Parp-1*^-/- ^cells, the majority (71%) of differentially expressed genes (153/17,664 genes) was down-regulated in the cells deficient in *Trrap*, a co-factor of histone acetyltransferase [39].

Parp-1 is able to modify histones and contributes to the opening of condensed highly ordered chromatin structures [40]. Furthermore, Parp-1 is a structural component of the transcriptionally repressed state of chromatin, and transcription is reported to be activated by auto-modification activity in an NAD-dependent manner [12]. Therefore, the roles of Parp-1 as a chromatin-modifying factor may contribute to maintenance of global gene expression during cell proliferation through mechanisms involving polyADP-ribosylation, protein-protein interaction, and poly(ADP-ribose)-protein interactions.

### Biological impact of *Parp-1 *deficiency on gene expression relating to differentiation

We observed genes involved in the pathway of extraembryonic tissue development, namely *H19, Sparc, Sox17, and Gata6*, are up-regulated in untreated *Parp-1*^-/- ^ES cells (Table 3). In addition, during differentiation of ES cells after withdrawal of LIF, expression of *H19 *as well as other trophoblast marker genes were further up-regulated in *Parp-1*^-/- ^ES cells compared to *Parp-1*^+/+ ^ES cells (Fig. 4B). We previously reported that the increase of trophoblast marker genes, *Plf, Prlpa*, and *Tcfap2 *was detected in untreated *Parp-1*^-/- ^ES clone (*p *< 0.05) using GeneSpring 4.2 [15]. In the present paper, these genes were not picked up by GeneSpring 6.1 using two *Parp-1*^-/- ^ES clones, probably because the criteria which we applied in this study were highly restricted and the expression level of the genes needed to be relatively high in at least one genotype. This is consistent with the fact that the gene expression changes associated with trophoblast induction were observed only in a subpopulation of ES cells by *in situ *hybridization [15]. In fact, *Plf *gene expression is not detectable in undifferentiated *Parp-1*^+/+ ^and *Parp-1*^-/- ^ES cells by RT-PCR (Fig. 4B). In contrast, the differentially expressed genes picked up in the present study are expected to be the representative genes affected in a large cell population. *H19 *is likely to be one of such genes in *Parp-1*^-/- ^ES cells.

The biological function of *H19 *RNA has not been fully understood yet. Several lines of evidence show that the *H19 *gene is involved in extraembryonic tissue development as briefly mentioned earlier. The homozygous mutant animals with a targeted deletion of the maternal *H19 *gene are viable and fertile and display an overgrowth phenotype of fetus and placentae compared with wild-type [41]. Mouse parthenogenetic embryos showing the monoallelic expression of the *H19 *gene exhibit functional defects in placentae [18], suggesting that the *H19 *gene may play an important role in the extraembryonic tissue development, especially in placentae.

Increased potential of *Parp-1*^-/- ^ES cells to differentiation into trophoblasts seemed to reflect preferential differentiation of *Parp-1*^-/- ^ES cells to trophoblasts triggered by LIF withdrawal, as shown in Fig. 4B. Early increase of *H19 *expression suggests that the *H19 *gene might act as an upstream regulator for the trophoblast differentiation pathway.

## Conclusion

These results suggest that *Parp-1 *is required to maintain transcriptional regulation of a wide variety of genes on a genome-wide scale. In *Parp-1*^-/- ^ES cells and livers, we observed that the majority of the altered genes were down-regulated. These down- and up-regulated genes are involved in a variety of cellular processes, including transcription, metabolism, signaling, immune response, cell structure, and other cellular processes. In this study, we showed that the pathway of extraembryonic tissues including trophoblast lineage is potentially up-regulated at an untreated state and after differentiation stimuli in *Parp-1*^-/- ^ES cells. The gene expression profiles in *Parp-1*-deficient cells may be useful to delineate the functional role of Parp-1 in epigenetic regulation of the genomes involved in various biological phenomena.

## Methods

### Cell lines and culture conditions

*Parp-1*^-/- ^ES cell clones, 210-58 and 226-47, established independently from *Parp-1*^+/- ^ES cells clones, 210 and 226, respectively, were used in this study [28]. They were all derived from male J1 ES cells. The ES cell lines were maintained in Dulbecco's modified Eagle's medium (Invitrogen) containing 20% fetal calf serum supplemented with amino acids and leukemia inhibitory factor (LIF), ESGRO (Chemicon) in the absence of a STO feeder, and total RNA was prepared as described below. Differentiation of ES cells by withdrawal of LIF was induced by inoculating 3 × l0^6 ^of ES cells in suspension in a culture dish (OPTILUX^® ^Petri dish, Becton Dickinson) containing 10 ml of ES medium without LIF. Medium was changed at days 3 and 5. At days 3, 5, and 7, all the cells including floating embryoid bodies were collected. The livers were prepared from *Parp-1*^+/+ ^and *Parp-1*^-/- ^female mice at 13 months of age [42], and about one-fifth of the amount of livers was used for total RNA extraction. Primary mouse embryonic fibroblasts (EFs) were derived from embryos at day 13.5 obtained by sister-brother mating of *Parp-1*^+/- ^mice with a 129Sv/ICR mixed genetic background as previously described [43]. Briefly, each embryo was minced, trypsinized, and dispersed cells were incubated for 1 or 2 days until the EF cells became confluent. The EF cells were replated on four dishes and when they became confluent, these EF cells were defined to be at the 3 population doubling level (PDL). When the EF cells reached 6 PDL, they were harvested when they reached half confluency.

### Total RNA isolation

Total RNA was extracted from ES cells, the livers, and EF cells using Isogen (Nippon Gene). Fifty micrograms of total RNA were treated with 5 units of DNase I (Invitrogen) for 15 min at room temperature, and purified again with Isogen.

### Oligonucleotide microarray

Sample preparation and microarray processing were carried out according to the protocol supplied by Affymetrix. Briefly, 5 *μ*g of total RNA sample treated with DNase I were reverse-transcribed by Superscript II reverse transcriptase (Invitrogen) using T7-(dT)_24 _primer containing T7 RNA polymerase promoter sequence. After second-strand complementary DNA (cDNA) synthesis, the product was used in an *in vitro *transcription reaction to generate biotinylated complementary RNA (cRNA) using a BioArray™ HighYield™ RNA Transcript Labeling Kit (Enzo Diagnostics, Inc). Fifteen micrograms of fragmented cRNA were hybridized to a murine genome U74A version 2 micro-array (Affymerix) for 16–18 hours at 45°C with constant rotation at 60 rpm. This high-density oligonucleotide microarray contained 12,488 mouse genes/EST.

After hybridization, the microarray was washed and stained with streptavidin R-phycoerythrin conjugate using an Affymetrix Fluidics Station. The fluorescence intensity was measured twice for each microarray and the average fluorescence intensity was normalized by global scaling to 1,000. The data were saved in Microsoft Excel files, then imported into a GeneSpring^® ^6.1 software database (Silicon Genetics). The data sets for J1 and 210-58 (*Parp-1*^-/-^) ES cells partially discussed in Hemberger *et al*. [15] were included in this study and further analyzed with GeneSpring^® ^6.1.

### Data analysis

Data analysis was performed with the GeneSpring^® ^6.1 software. For statistical analyses, the fluorescence intensity (raw signal) was normalized to the median reading per chip, and then normalized to median reading per gene.

We used 6 replicates for each non-parametric tests with the global standard error model being inactive because more than five replicates were recommended for the tests. In the case of *Parp-1*^-/- ^ES cells, 6 replicates consisting of triplicate microarray results from two *Parp-1*^-/- ^ES cell lines were used. In the case of livers, 6 replicates consisting of triplicates obtained from two different animals, respectively, were used for each genotype. In the case of EF cells, 3 replicates obtained using three different embryos were used for each genotype and the global standard error model was active. We excluded those genes that showed a standard deviation greater than 2.0 in the normalized data of both genotypes, therefore, we started analysis with 9,907, 12,353, and 12,359 genes and ESTs for ES cells, livers, and EFs, respectively (Table 1). We constructed gene lists only with the genes that showed statistical differences (*p *< 0.05 or *p *< 0.01) and 2-fold or greater differences in normalized expression levels between *Parp-1 *genotypes. To construct heatmaps, we used GeneSpring^® ^GX ver. 7.3.1 (the latest version).

### Northern blot analysis

Total RNA samples (10 *μ*g) were used for northern blot analysis as described elsewhere [15]. We used the 90 bp (*Igfbp3*) or the 89 bp (*Galnt1*) cDNA fragment as a probe. The membrane was hybridized with the probe and was washed. The membrane was exposed to a Fuji Imaging Plate (Fuji film), and the radioactivities were analyzed using BAS-2500 Bio-imaging analyzer (Fuji film).

### Reverse transcription polymerase chain reaction (RT-PCR)

We used Superscript™ III First-Strand Synthesis System for RT-PCR kit (Invitrogen). First-strand cDNA was synthesized from 2 *μ*g each of DNase I-treated total RNA using an oligo(dT)_20 _primer and Superscript™ III reverse transcriptase. After the first-strand cDNA synthesis, PCR amplification was performed using TAKARA Ex *Taq *(Takara Bio) with primers listed in Table S1 (see Additional file 1). The thermal cycle conditions were as follows: 94°C for 2 min, then 18 cycles (*Oct3/4*), 20 cycles (*Gapdh*), 22 cycles (Fig. 4B) or 24 cycles (Fig. 4A) (*H19 *and *Igf2*). For *Cdx2*, 30 cycles at 94°C for 30 sec, 60°C for 30 sec, and 72°C for 30 sec were carried out. For *Plf*, 94°C for 2 min, then 40 cycles at 94°C for 30 sec, 68°C for 2 min 30 sec, and then 72°C for 3 min. Products were run on 1.5–3% agarose gel and stained with ethidium bromide. Confirmation of PCR products was carried out by direct sequencing.

## Authors' contributions

HO, TN, TO, M. Maeda, HS, YM, HN, and M. Masutani designed the experiments. HO, TN, AG, M. Maeda, and M. Masutani performed the experiments. HO and M. Masutani prepared the manuscript. HS contributed to maintaining *Parp-1 *knockout mice. M. Masutani, HN, and TS coordinated the project.

## Supplementary Material

Additional File 1 Table S1. Primers used in this study. Primers used in RT-PCR analysis (Fig. 4). Click here for file

## Figures and Tables

**Table 1:** Differential expression of genes between *Parp-1*^+/+ ^and *Parp-1*^-/- ^ES cells, livers, and EFs

	No. of genes				
	
		*Parp-1*^-/- ^<*Parp-1*^+/+^		*Parp-1*^-/- ^> *Parp-1*^+/+^	
			
*p*-value cut off^a^	Total	Total	2-fold or greater	Total	2-fold or greater
*ES cells*^c^					
Total^b^	9,907	5,464	1,283	4,349	1,406
*p *< 0.05^b^	2,273	1,609	626	664	324
*p *< 0.01^b^	928	684	259	244	120
*Livers*^d^					
Total^b^	12,353	7,138	1,184	4,860	1,038
*p *< 0.05^b^	1,616	1,190	253	426	158
*p *< 0.01^b^	641	515	100	126	43
*EFs*^e^					
Total	12,359	5,042	707	7,317	501
*p *< 0.05	996	390	216	606	205

^a ^Analyzed by One-Way ANOVA (non-parametric test known as Wilcoxon-Mann-Whitney test)

^b ^These genes were presented in Fig. 1 (A)-(F).

^c ^*Parp-1*^+/+ ^ES cell clone, J1, and *Parp-1*^-/- ^ES cell clones, 210-58 and 226-47, were used.

^d ^Two mice were used for each genotype.

^e ^Three EFs obtained from three embryos were analyzed as triplicate experiments.

**Table 2:** Genes down-regulated in *Parp-1*^-/- ^ES cells

	Fold change^a)^					
				
Accession No.	W vs H	J1 vs 210-58	J1 vs 226-47	Symbol	Chromosome	Gene description
**Cell cycle/cell proliferation/cell death**						
AW122355	3.2	5.2	2.3	*Prkcbp1*	2	Protein kinase C binding protein 1
AF067395	2.9	2.9	2.9	*Bnip3l*	14	BCL2/adenovirus E1B 19 kDa-interacting protein
AI842277	2.7	2.3	3.2	*Igfbp3*	11	Insulin-like growth factor binding protein 3
U95826	2.2	2.5	1.9	*Ccng2*	5	Cyclin G2
**Cell structure/cell adhesion**						
U16741	4.1	6.3	3.1	*Capza2*	6	Capping protein (actin filament) muscle Z-line, alpha 2
AI132380	3.6	3.1	4.3	*Fndc3a*	14	Fibronectin type III domain containing 3a
AI505453	2.9	2.5	3.4	*Myh9*	15	Myosin, heavy polypeptide 9, non-muscle
AW208938	2.4	3.2	2.0	*Pkp2*	16	Plakophilin 2
M76124	2.4	2.2	2.6	*Tacstd1*	17	Tumor-associated calcium signal transducer 1
**Metabolism**						
U73820	5.5	5.2	5.8	*Galnt1*	18	Polypeptide GalNAc transferase-T1 (ppGaNTase-T1)
AI841270	3.4	2.4	6.4	*Gstm1*	3	Glutathione S-transferase, mu1
AV308550	2.6	4.1	1.9	*Piga*	x	Phosphatidylinostitol glycan, class A
AI851912	2.3	2.2	2.5	*Rps27*	3	Ribosomal protein S27
AI852144	2.1	2.9	1.7	*Pbef-pending*	12	Pre-B-cell colony-enhancing factor
U65986	2.1	1.9	2.5	*Anxa11*	14	Annexin A11
D50264	2.1	1.4	4.1	*Pigf*	17	Phosphatidylinositol glycan, class F
AF031486	2.0	2.0	2.0	*Sms*	x	Spermidine synthase
AI845882	2.0	2.5	1.7	*Acyp1*	12	Acylphosphatase1, erythrocyte (common) type
**Protein biosynthesis/degradation**						
AI852581	3.0	3.0	3.1	*Ide*	19	Insulin degradating enzyme
AI414051	3.0	1.8	9.1	*Usp24*	4	Ubiquitin specific protease 24
AW121012	2.9	2.8	2.8	*Rnf19*	15	Ring finger protein 19
X92665	2.9	2.5	3.4	*Ube2e1*	14	Ubiquitin-conjugating enzyme UbcM3
AW048882	2.2	2.8	1.8	*Iars*	13	Isoleucine-tRNA synthetase
AA867340	2.2	1.9	2.6	*Psme4*	11	Proteasome (prosome, macropain) activator subunit
AB024427	2.2	2.3	2.1	*Rnf11*	4	Ring finger protein 11
**Signaling**						
AI846023	4.6	2.8	13.1	*Arl7*	1	ADP-ribosylation factor-like 7
AA260005	2.8	2.7	2.8	*Pawr*	10	PPKC, apoptosis, WT1, regulator
AI317205	2.6	2.4	2.7	*Map3k1*	13	Mitogen activated protein kinase kinase kinase 1
AF035644	2.3	2.0	2.7	*Ptp4a2*	4	Protein tyrosine phosphatase 4a2
M21019	2.3	1.9	2.9	*Rras*	7	Harvey rat sarcoma oncogene, subgroup R
AI194248	2.2	2.5	1.9	*Csnk2a1*	2	Casein kinase II, alpha 1 polypeptide
AI854006	2.0	2.0	2.1	*Set*	2	SET translocation
D83921	2.0	1.9	2.1	*Ebaf*	1	Endometrial bleeding associated factor
**Transcription/replication**						
X14206	9.9	8.4	12.0	*Adprt1*	1	Poly(ADP-ribose) polymerase 1
M99167	3.0	6.2	2.0	*Hnrpa1*	15	Heterogeneous nuclear ribonucleoprotein A1
AW107922	2.8	3.7	2.2	*Sox11*	12	SRY box-containing gene 11
AI849135	2.5	2.5	2.5	*Foxo3a*	10	Forkhead box 03a
Y07836	2.5	2.3	2.8	*Bhlhb2*	6	Basic-helix-loop-helix domain containing, class B2
X74760	2.5	2.3	2.7	*Notch3*	17	Notch gene homolog 3, (Drosophila)
AI447783	2.1	2.4	1.9	*Helb*	10	Helicase(DNA) B
X94694	2.1	2.7	1.7	*Tcfap2c*	2	Transcription factor AP-2, gamma
AF077861	2.1	2.2	2.1	*Id2*	12	Inhibitor of DNA binding 2
AI605405	2.0	1.9	2.2	*Phf13*	4	PHD finger protein 13
D78382	2.0	1.7	2.6	*Tob1*	11	Transducer of ErbB2.1
**Transport**						
AV356315	4.1	5.5	3.3	*Lman1*	18	Lectin, mannose-binding, 1
AV298789	2.9	2.6	3.2	*Ranbp5*	14	Ran binding protein 5
D88315	2.2	2.2	2.2	*Hiat1*	3	Hippocampus abundant gene transcript 1
**Unknown**						
AI845617	3.5	3.5	3.4	*2610019A05Rik*	11	Hypothetical protein
AI852287	3.2	3.3	3.2	*Ankrd28*	14	Ankyrin repeat domain 28
AI836771	3.0	2.8	3.3	*2900008M13Rik*	15	Unknown EST
AA684456	2.9	2.1	4.5	*2310015N07Rik*	7	Hypothetical protein
AI848435	2.8	1.9	4.8	*C78339*	13	Unknown EST
AW123157	2.7	2.5	3.1	*1700051E09Rik*	11	Hypothetical protein
AW124843	2.6	3.1	2.3	*C85108*	4	Unknown EST
AA710439	2.6	2.0	3.6	*6230421P05Rik*	16	Unknown EST
AI853444	2.5	1.8	3.9	*2610042L04Rik*	14	Hypothetical protein
AI853444	2.2	2.1	2.3	*2610042L04Rik*	14	Hypothetical protein
AW121353	2.1	1.6	3.1	*Lrrc8*	2	Luecine rich repeat containing 8
AI037493	2.1	1.5	3.4	*Tbc1d15*	10	TBC1 domain family, member 15
AI461803	2.1	2.2	1.9	*1300006C19Rik*	9	Hypothetical protein
AW049969	2.0	2.0	2.1	*C330005L02Rik*	9	Hypothetical protein
AI847483	2.0	2.0	2.0	*Tmem41b*	7	Transmembrane protein 41B

^a)^W, wild-type cells (J1); H, *Parp-1*^-/- ^ES cells (210-58 and 226-47).

**Table 3:** Genes up-regulated in *Parp-1*^-/- ^ES cells

	Fold change^a)^					
				
Accession No.	H vs W	210-58 vs J1	226-47 vs J1	Symbol	Chromosome	Gene description
**Cell cycle/cell proliferation/cell death**						
X58196	3.1	3.3	2.9	*H19*	7	H19 non-coding RNA
AI842665	3.0	3.1	2.8	*Tax1bp3*	11	Human T-cell leukemia virus type I binding protein 3
**Cell structure/cell adhesion**						
X04017	2.3	2.3	2.3	*Sparc*	11	Cysteine-rich glycoprotein SPARC
M26071	2.1	2.5	1.8	*F3*	3	Coagulation factor III
M91236	2.1	2.1	2.1	*Gjb5*	4	Gap junction membrane channel protein beta 5
**Immune response**						
U13705	2.3	2.1	2.4	*Gpx3*	11	Glutathione peroxidase 3
**Metabolism**						
AW120625	2.3	1.9	2.7	*Pgd*	4	Phosphogluconate dehydrogenase
M64782	2.2	1.9	2.5	*Folr1*	7	Folate-binding protein 1 (FBP1)
X97755	2.0	2.1	2.0	*Ebp*	x	Phenylalkylamine Ca2+ antagonist (emopamil) binding protein
**Protein biosynthesis/degradation**						
W71352	3.9	4.2	3.6	*Bag2*	1	Bcl2-associated athanogene 2
AI844175	3.4	3.4	3.4	*Mrps11*	7	Mitochondrial ribosomal protein S11
U16163	2.9	2.9	2.8	*P4ha2*	11	Prolyl 4-hydroxylase alpha(II)-subunit
D00622	2.5	2.0	3.0	*Lrpap1*	5	Low density lipoprotein receptor related protein, associated protein 1
X60676	2.3	2.4	2.2	*Serpinh1*	7	HSP47
AW124432	2.1	1.8	2.5	*Mrpl12*	11	Mitochondrial ribosomal protein L12
AI839392	2.0	2.0	2.1	*Aars*	8	Alanyl-tRNA syntase
**Transcription/replication**						
D49473	3.4	3.0	3.7	*Sox17*	1	SRY-box containing gene 17
U51335	2.5	2.5	2.6	*Gata6*	18	GATA-binding protein 6
U79962	2.4	2.1	2.6	*Tarbp2*	15	TAR (HIV) RNA binding protein 2
D49473	2.1	1.9	2.3	*Sox17*	1	SRY-box containing gene 17
**Transport**						
D14077	2.2	2.1	2.2	*Clu*	14	Clusterin
**Others**						
M34603	2.6	2.3	3.0	*Prg*	10	Proteoglycan core protein
AA793009	2.3	2.0	2.7	*Tex19*	11	Testis expressed gene 19
**Unknown**						
AI846553	3.2	3.0	3.3	*1110020C13Rik*	15	Hypothetical protein
AI845664	2.1	2.0	2.2	*Grwd*	7	Glutamate-rich WD repeat containing 1

^a) ^H, *Parp-1*^-/- ^ES cells (210-58 and 226-47); W, wild-type cells (J1).

**Table 4:** Genes down- and up-regulated in *Parp-1*^-/- ^livers

		Fold change^a)^							
					
	Accession No.	W vs H	W1 vs H1	W1 vs H2	W2 vs H1	W2 vs H2	Symbol	Chromosome	Gene description
**Down-regulated**									
**Cell structure/cell adhesion**									
	AA867778	2.1	2.4	2.6	1.7	1.8	*Actn1*	12	Actinin, alpha 1
**Cell cycle/cell proliferation/cell death**									
	AJ223782	2.0	1.8	1.7	2.5	2.3	*Sept7*	9	Septin7 (Cdc10)
**Immune response**									
	X05475	2.1	2.5	1.8	2.6	1.9	*C9*	15	Complement component C9
**Metabolism**									
	L42996	3.0	1.7	3.7	2.7	5.8	*Dbt*	3	Nuclear-encoded mitochondrial acyltransferase
	AF026075	2.4	1.8	4.3	1.7	4.0	*Sult3a1*	10	Sulfotransferase-related protein (SULT-X2)
**Protein biosynthesis/degradation**									
	M27347	3.2	3.4	3.2	3.1	3.0	*Ela1*	15	P6-5 gene, 3' end (elastase 1)
**Signaling**									
	AI563623	2.3	2.9	1.9	2.9	1.8	*Pkn2*	3	Protein kinase N2
**Transcription/replication**									
	AF010405	4.9	6.8	3.2	8.5	4.1	*Hfh-1L*	13	HNF-3/forkhead homolog 1 like
	L20450	3.7	3.1	2.7	5.0	4.3	*Zfp97*	17	Zinc finger protein 97
	AW048355	2.1	1.6	1.9	2.3	2.8	*Phf17*	3	PHD finger protein 17
	AI848996	2.1	2.2	2.3	2.0	2.1	*Dhx40*	11	DEAH box polypeptide 40
	AW123909	2.1	1.5	1.9	2.2	2.9	*Rbpms*	8	RNA binding protein gene with multiple splicing
**Transport**									
	D86066	3.2	2.3	4.4	2.6	4.8	*Rab5ep-pending*	11	Rabaptin-5
**Others**									
	AI835016	2.4	2.1	2.3	2.5	2.7	*Hps4*	5	Light ear protein (1e)
**Unknown**									
	AI848841	2.1	2.2	1.6	2.7	2.0	*A230106A15Rik*	13	Unknown

**Up-regulated**		H vs W	H1 vs W1	H1 vs W2	H2 vs W1	H2 vs W2			
					
**Cell cycle/cell proliferation/cell death**									
	X95280	3.0	2.8	2.7	3.4	3.2	*G0s2*	1	GOS2-like protein
**Cell structure/cell adhesion**									
	AI132491	2.1	1.9	2.6	1.6	2.2	*Bysl*	17	Bystin-like
**Immune response**									
	J00475	3.1	9.2	2.8	4.2	1.3	*Iga*	12	Germline IgH chain gene, DJC region-segment D-FL16.1
**Metabolism**									
	M63245	3.2	2.8	4.0	2.6	3.7	*Alas1*	9	Amino levulinate synthase (ALAS-H)
	AW121625	2.5	2.8	2.4	2.6	2.3	*Galnt11*	5	Polypeptide GalNAc transferase 11
	Y15003	2.1	1.8	1.9	2.3	2.5	*St3gal5*	6	Beta-galactoside alpha-2,3-sialyltransferase 5
**Signaling**									
	L76567	4.1	1.8	2.3	5.5	7.0	*Shp1*	4	Shp gene
**Transcription/replication**									
	AI553024	2.4	2.4	1.5	3.8	2.4	*Zbtb16*	9	Zinc finger and BTB domain containing 16
**Unknown**									
	AI042964	7.1	7.1	8.4	5.9	7.1	*0610005C13Rik*	7	Hypothetical protein
	AI593759	3.7	3.0	4.0	3.4	4.6	*9530051K01Rik*	7	Hypothetical protein
	AI019679	2.3	10.0	1.4	9.4	1.3	*1100001G20Rik*	11	Hypothetical protein

^a) ^W, *Parp-*^+/+ ^livers from two animals (W1 & W2): H, *Parp-1*^-/- ^livers from two animals (H1 & H2).
