# Supplementary material for: Loss of Parp-1 affects gene expression profile in a genome-wide manner in ES cells and liver cells
Source: BMC Genomics. 2007 Feb 7;8:41. doi: 10.1186/1471-2164-8-41 (PMC1810537; doi:10.1186/1471-2164-8-41)
Supplement: Additional File 1 — Table S1. Primers used in this study. Primers used in RT-PCR analysis (Fig. 4). [file 1471-2164-8-41-S1.pdf]

Table S1. Primers used in this study

Gene	Forward primer (5'-3')	Reverse primer (5'-3')	Product size (bp)	Annealing temperature (ºC)	Cycle no.	Reference
H19	CATGTCTGGGCCTTTGAA	TTGGCTCCAGGATGATGT	245	60	22, 24	1
Igf2	AGGGGAGCTTGTTGACACG	GGGTATCTGGGGAAGTCGTC	225	60	22, 24	1
Cdx2	AGGCTGAGCCATGAGGAGTA	TGAGGTCCATAATTCCACTCA	114	60	30	2
Plf	CTCTGCAGAGATGCTCCCTTC	CATGATATTTCAGAAGCAGAGCAC	721	68	40	3
Oct 3/4	CAACGAGAAGAGTATGAGGC	AAGCTCCAGGTTCTCTTGTC	267	60	18	This study
Gapdh	TGGTGAAGGTCGGTGTGAAC	AGGGGTCGTTGATGGCAACA	103	60	20	This study

1. Ogawa H, Ono Y, Shimozawa N, Sotomaru Y, Katsuzawa Y, Hiura H, Ito M, Kono T: **Disruption of imprinting in cloned mouse fetuses from embryonic stem cells**. *Reproduction* 2003, **126**:549-557.
2. Niwa H, Toyooka Y, Shimosato D, Strumpf D, Takahashi K, Yagi R, Rossant J: **Interaction between Oct3/4 and Cdx2 determines trophectoderm differentiation.** *Cell* 2005, **123**:917-929.
3. Fang Y, Lepont P, Fassett JT, Ford SP, Mubaidin A, Hamilton RT, Nilsen-Hamilton M: **Signaling between the placenta and the uterus involving the mitogen-regulated protein/proliferins.** *Endocrinology* 1999, **140**:5239-5249.
